# Dynamics and universal scaling law in geometrically-controlled sessile drop evaporation

**DOI:** 10.1038/ncomms14783

**Published:** 2017-03-15

**Authors:** P. J. Sáenz, A. W. Wray, Z. Che, O. K. Matar, P. Valluri, J. Kim, K. Sefiane

**Affiliations:** 1 Department of Mathematics, Massachusetts Institute of Technology, Cambridge, Massachusetts 02139, USA; 2 Department of Chemical Engineering, Imperial College London, London SW7 2AZ, UK; 3 School of Engineering, The University of Edinburgh, Kings Buildings, Mayfield Road, Edinburgh EH9 3FB, UK; 4 Department of Mechanical Engineering, The University of Maryland, College Park, Maryland 20742, USA; 5 Tianjin Key Lab of Refrigeration Technology, Tianjin University of Commerce, Tianjin City 300134, China

## Abstract

The evaporation of a liquid drop on a solid substrate is a remarkably common phenomenon. Yet, the complexity of the underlying mechanisms has constrained previous studies to spherically symmetric configurations. Here we investigate well-defined, non-spherical evaporating drops of pure liquids and binary mixtures. We deduce a universal scaling law for the evaporation rate valid for any shape and demonstrate that more curved regions lead to preferential localized depositions in particle-laden drops. Furthermore, geometry induces well-defined flow structures within the drop that change according to the driving mechanism. In the case of binary mixtures, geometry dictates the spatial segregation of the more volatile component as it is depleted. Our results suggest that the drop geometry can be exploited to prescribe the particle deposition and evaporative dynamics of pure drops and the mixing characteristics of multicomponent drops, which may be of interest to a wide range of industrial and scientific applications.

## Abstract (web summary)

Drop evaporation can be used as a fabrication technology for targeted particle deposition or microflow control, yet previous research is limited to spherical drops. Here, Sáenz *et al*. generalize the evaporation dynamics for arbitrary drop geometry and show its potential for more sophisticated control.

Previous literature studies of liquid droplets resting on solid substrates undergoing evaporation have focused on spherical configurations in order to take advantage of axisymmetry^1^,^2^,^3^. This is in an effort to ameliorate the complexity of the evaporation process related to a delicate interaction between the bulk flow^4^,^5^,^6^,^7^,^8^, evaporation kinetics^9^,^10^,^11^, thermocapillary stability^12^,^13^ and binary-mixture dynamics^14^,^15^,^16^. The flow is complicated further by the presence of moving contact lines as well as regions of high curvature, both of which influence the evaporation.

In what follows, we revisit these milestone investigations^4^,^10^,^12^,^14^ of small spherical sessile drops (spatially unique contact angle) and extend their scope to the unexplored case of non-spherical geometries (spatially varying contact angle). This enables us to demonstrate the tremendous loss of physical richness accompanying the simplifying axisymmetric assumption. Our results, which elucidate the strong interplay between the geometry and flow dynamics, may pave the way for the development of novel, cheap and versatile micro- and nano-fabrication technologies in a wide range of applications; these include targeted colloidal deposition (bio-microarrays for protein and DNA sequencing^17^, inkjet printing^18^, metal printing^19^) or microflow control (DNA stretching^10^).

## Results

### Experimental methods

In order to generate reproducible non-spherical sessile drops with well-defined geometries, we machined and polished very thin pedestals (height *H*=0.1±0.05 mm) with given canonical shapes (including circular, pentagonal, square and triangular) and more exotic geometries (for example, kidney or half-moon shapes) in aluminium disks with a large thermal mass (diameter × height=3.8 × 1 cm). In some experiments, the substrates were heated and kept at constant temperature *T*_w_ by means of a film heater attached to the lower side, a thermocouple and a proportional-integral-derivative feedback loop. The size of the pedestals shapes was chosen such that the contact line would have a predetermined perimeter *P* equal for all of them. Three sizes were considered: *P*=12, 15 and 17 mm. Sharp corners were purposely avoided as these would correspond to singularities according to the Laplace pressure. The corner radius was selected to be *R*_c_=0.4 mm in all cases. A given volume of liquid *V*⩽7 μl (±1.2%) placed on such geometries with a micropipette would be pinned at the edges of the pedestal, thus leading to highly reproducible drop shapes. Each experiment was repeated at least five times. Drops of pure water required additional treatment of the pedestal's upper surface with a hydrophilic coating prior to drop deposition to ensure precise, repeatable contact-line pinning. Tests were carried out to ensure that the effect of the pedestal height was negligible, and that the coating did not have any influence on the flow dynamics or evaporation kinetics (see Methods).

The resulting drops remained pinned for most of their lifetime (>70%). The drop mass, *m*, was instantaneously measured with a microbalance (readability 0.01 mg) while the drops evaporated in a controlled chamber; the instantaneous evaporation rate is, thus, defined as 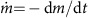. Infrared thermography, as described elsewhere^20^, was used to observe the drop's thermal field. In all cases considered in the present work, the drop height (maximum 1.1 mm) was always smaller than the capillary length 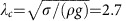 mm with water and 1.7 mm with ethanol. Here *σ*, *ρ* and *g* denote surface tension, liquid density and gravitational acceleration, respectively. Hence, surface tension dominates gravity and, thus, the interface shape results from the surface energy minimization of the system with the constraint that the contact line is pinned along the prescribed geometry on the substrate.

### Evaporation kinetics in quasi-isothermal drops

**Figure 1:**
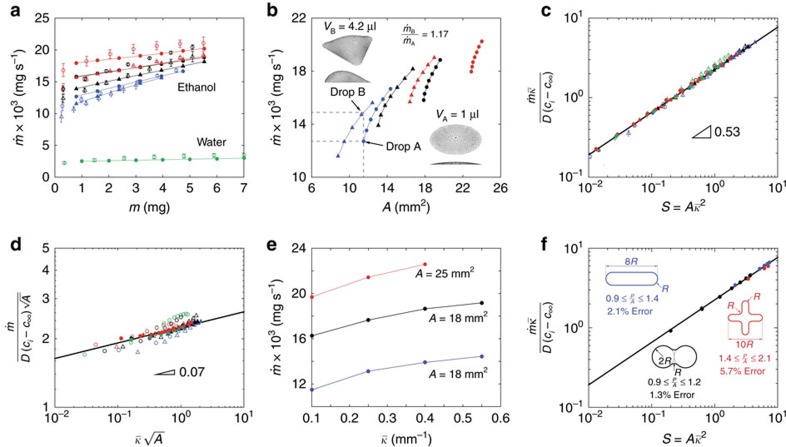
Evaporation kinetics of non-heated sessile drops. (**a**) Experimental (hollow symbol) and numerical (filled symbol+solid line) evaporation rates for sessile drops with circular (circular symbol) and triangular (triangle) contact areas of different perimeter lengths *P* and volume 1⩽*V*⩽7 μl. Blue corresponds to *P*=12 mm, black and green to *P*=15 mm and red to *P*=17 mm. The error bars represent the s.d. of the experimental measurements. The room temperature and humidity were maintained constant at *T*_a_=23 °C and RH=50%, respectively. The numerical evaporation rate is 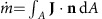 where **J**=−*D*∇*c* is the local evaporation flux, *A* the interfacial area, **n** the unit vector normal to the interface, *D* the molecular diffusivity and *c* the vapour concentration resulting from steady-state diffusion in the gas, that is, ∇^2^*c*=0, with appropriate boundary conditions. A detailed description of the model is available in the Methods section. (**b**) Numerical evaporation rates (extracted from **a**) versus liquid–gas surface area *A* illustrating how two drops with the same *P* and *A* but different shape lead to significantly different evaporation rates. (**c**) Scaling law including the area-average interface mean curvature 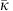 that collapses all the data shown in **a**. (**d**) Re-plotting of the data and the scaling law to illustrate the effect of 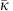. (**e**) Numerical evaporation rates for different spherical drops with the same *A* but varying 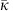 in the range of the characteristic values examined experimentally. (**f**) Numerical simulations testing the scaling law performance in more ‘exotic' shapes with *P*=15 mm and varying *V*.

We start by considering the quasi-isothermal case, in which the substrate remained at room temperature, in order to examine the evaporation kinetics^5^,^9^,^10^,^11^ of non-spherical drops. We considered drops of pure liquids with relatively low volatility (ethanol and distilled water). Under such conditions, the timescale of diffusion of vapour in the gas *O*(1 s) is much shorter than the evaporation timescale *O*(100 s)^1^. Since quasi-isothermal conditions also ensure negligible buoyancy-driven convection currents in the gas, the evaporation kinetics of these drops are the result of (quasi) steady-state diffusion in the gas, that is, ∇^2^*c*=0, where *c* is the vapour concentration in the gas phase. We thus performed a set of numerical simulations solving the steady-state vapour diffusion problem with appropriate boundary conditions (see Methods), and compared the results with the experimental values for different drop sizes (Fig. 1a). In these simulations, the three-dimensional (3D) drop shapes were calculated with Surface Evolver^21^ and entered the problem as part of the lower gas-domain boundary. Only circular and triangular contact shapes are presented since these are the limiting cases for regular polygons. The evaporation rates of other canonical geometries, for example, pentagonal shape, fall between the curves for the circular and triangular geometries. The overall agreement is very good, considering the purposely sought simplicity of the model, which corroborates that this is essentially a diffusion-limited phase change process.

Both the experiments and the numerical simulations demonstrate that the evaporation rate 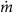 increases with *P*, and that for two drops with the same *V* and *P*, the spherical drop evaporates faster than the triangular. By re-plotting the data to demonstrate the dependence of 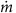 on the interfacial liquid–gas area, *A*, as shown in (Fig. 1b), we illustrate a key observation: drops of different shape with the same *P* and *A* give rise to a significantly different 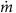. Note the inversion here: triangular drops evaporate faster than circular drops with the same *A*. In the particular example shown, 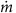 changes by 17%. Hence, one cannot expect to find a general scaling law of the form 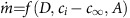 as may have seemed logical. Here *D* is the molecular diffusivity, and *c*_*i*_−*c*_∞_, the difference between the interface and the far-field gas concentrations. In other words, this observation demonstrates the invalidity of the classic diffusion-limited scaling argument, which suggests that 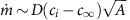. The general scaling law must incorporate a shape factor that accounts for the drop geometry. We thus included the area-averaged mean interface curvature 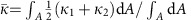, where *κ*_*i*_(*i*=1, 2) corresponds to the principal radii of curvature, which led to the data collapse depicted in Fig. 1c with:


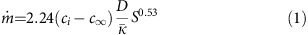


where *S*=*A*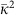 is a dimensionless shape factor. The average absolute error between the scaling law and the data is 1.08%. It should be noted that curvature of the experimental drops was inferred through Surface Evolver, which was supported by the agreement found between the experimental and theoretical drop profiles as shown in the Methods section. The scaling relation (1) captures the essence of the central result presented in Fig. 1b: the interface curvature enhances evaporation. In other words, a more rounded drop (drop B) evaporates faster than a flatter drop with the same *A* and *P* (drop A). The enhancing effect of 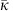 in the evaporation rate becomes evident by noting that 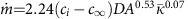 or by re-plotting the evaporation data as shown in Fig. 1d. The origin of the exponents is at the moment an open question, but one must expect that in the limit when *m*→0 the problem becomes independent of 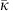 since the final evaporation rate in pinned drops is finite^9^. It is also important to realize that this effect is equally relevant even when only spherical drops are considered, see Fig. 1e. Finally, we also tested numerically the range of applicability of (1) in Fig. 1f and found that the average error increases slightly with the *P*/*A* ratio.

**Figure 2:**
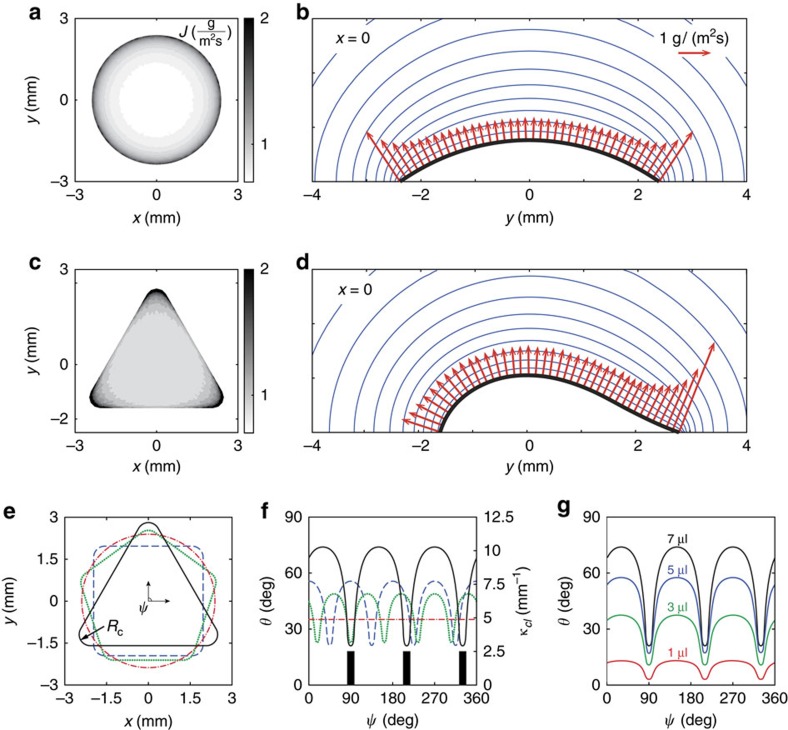
Simulated evaporation flux distribution and drop geometry. Evaporation flux **J** distribution in a 7 μl drop with (**a**,**b**) circular and (**c**,**d**) triangular contact area, and *P*=15 mm for the ethanol drops described in Fig. 1. (**e**) Prescribed contact-line geometry for the canonical shapes with *P*=15 mm and *R*_c_=0.4 mm. (**f**) Corresponding contact-angle *θ* distribution with *V*=7 μl along with the local contact-line curvature *κ*_cl_ for the triangular drop (filled bars) in terms of the azimuthal angle *ψ* as defined in **e**. The rest of the *κ*_cl_ maps are not shown for the shake of clarity. (**g**) Contact-angle distribution in the triangular geometry for varying drop volume.

The same simple model allows us to illustrate the local distribution of the evaporation flux **J**=−*D*∇*c* as a function of the drop shape. In drops with circular contact area (Fig. 2a,b), this is axially symmetric and rather uniform along the interface except for near the contact line where it spikes^4^,^10^. As the contact angle *θ*→0°, the contact-line flux spike becomes even more pronounced. This results from the drop geometry combined with the non-penetration boundary condition at the substrate. Iso-concentration curves, which are almost parallel to the interface just above the drop, approximate closer to the horizontal at low contact angles and, thus, become more abruptly curved in order to fulfil the non-penetration (90°) condition at the solid substrate. This effect leads to larger *c* gradients emerging near the contact line and, consequently, to enhanced evaporation fluxes in the same regions. In the opposite limit, hemispherical drops (*θ*=90°) result in a constant evaporation flux everywhere.

In the case of a triangular drop (Fig. 2c,d), **J** is found to additionally vary along the contact line, the maxima being located at the base apices. This problem is more complex and explaining the inhomogeneous **J** distribution involves not only the contact angle *θ* (that is, geometry effects in the vertical direction) but also the contact-line curvature *κ*_cl_ (geometry effects in the horizontal direction). We start by addressing the geometry effects in the vertical direction, that is, those associated with *θ*. As opposed to the case of spherical drops where *θ* is constant, non-spherical drops lead to varying *θ* distributions. Figure 2e shows the prescribed canonical contact-line geometries with *P*=15 mm, and Fig. 2f the corresponding *θ* and *κ*_cl_ maps with *V*=7 μl. Note that in pinned drops the *θ* distribution along the triple line is inversely proportional to *κ*_cl_ (ref. 20). In other words, ‘corners' in the contact line (large *κ*_cl_) correspond to areas of small *θ* (and consequently smaller local liquid film thickness that approximate more to the horizontal), and *vice versa*. Following the same rationale as for the spherical drop, the iso-concentration contours are thus more deformed at the base apices in order to meet the non-penetration condition at the adjacent substrate (Fig. 2d). Consequently, this leads to more pronounced *c* gradients in the vicinity of the apices and thus to a larger **J**, while the same effect is less pronounced in the rest of the contact line (where *θ* is larger). Figure 2g shows the evolution of the *θ* distributions for decreasing *V*. As the drop evaporates, *θ* becomes more uniform; there is a smaller deviation between the extreme values of *θ*, which indicates that the inhomogeneity in **J** due to variable *θ* tends to disappear towards the end of the drop lifetime. This, however, does not mean that **J** becomes uniform as *V*→0 μl. We also performed simulations of the triangular planar case (*V*=0 μl) and observed that the **J** distribution is qualitatively the same as that shown in Fig. 2c. Note that such inhomogeneous evaporation flux distribution cannot be explained in terms of varying *θ*, given that in these drops *θ*=0° everywhere.

We use this observation to introduce the second geometry effect playing a role (this in the horizontal direction and thus associated with *κ*_cl_) that leads to the azimuthally inhomogeneous **J** distribution observed. In the planar case the iso-concentration lines just above and outside the triangular contact area are parallel to the contact line and, at the apices, their shape need to be curved by 60° in order to align with their adjacent straight sides. Hence, in a similar manner to the vertical case, this abrupt geometrical readjustment enforced by the boundary conditions leads to larger *c* gradients at the apices and, therefore, to locally larger **J** fluxes. This explains why the contact-line corners display enhanced evaporation even in the planar case. Note that this effect becomes more prominent for increasing *κ*_cl_: as *κ*_cl_ becomes larger, the apex resembles more closely a sharp corner and so **J** is stronger. In the opposite case of a circular contact area, since *κ*_cl_ is constant, this effect does not result in any inhomogeneity in the azimuthal **J** distribution. It should be noted that these two geometrical effects that lead to enhanced **J** at the apices, one of which is associated with *θ* and the other with *κ*_cl_, act simultaneously when *V*>0 μl. In reality, they are the same effect and result from the ‘equi-dimensionality' of Laplace's equation. Note that *θ* can be seen as a measure of interfacial deformation but in the vertical direction. In summary, we conclude that locally **J** varies directly with *κ*_cl_ and inversely with *θ*.

### Coffee ring effect

**Figure 3:**
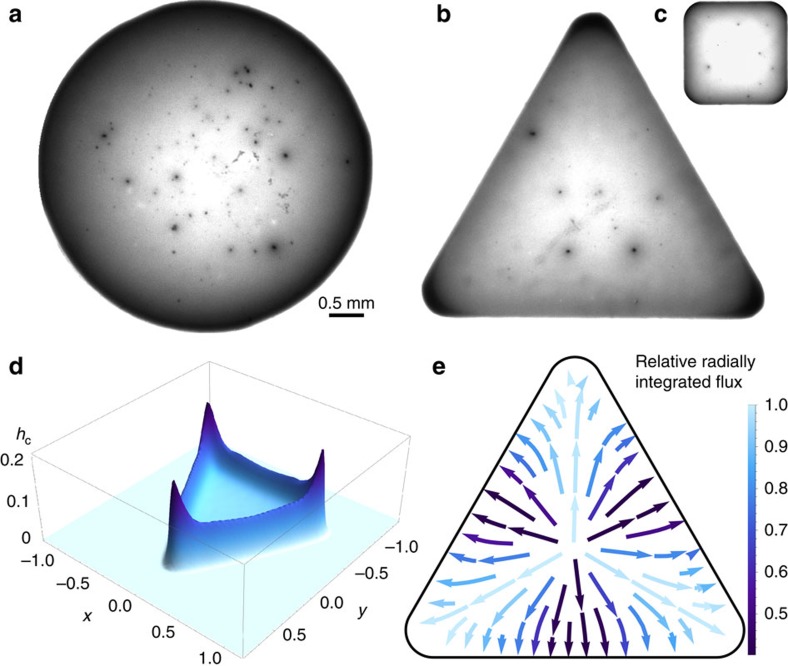
Coffee ring effect in non-spherical drops. Stains left by (**a**) a spherical and (**b**,**c**) non-spherical sessile drops of coffee evaporating in a closed chamber at room temperature. The initial volume is *V*=7 μl and the contact-line perimeter is *P*=15 mm in all cases. Non-spherical drops exhibit preferential particle deposition in contact-line regions where the curvature is higher, which contrast with the uniform ring observed with spherical drops. (**d**) Particle deposition distribution and (**e**) flow field calculated numerically for the same drop as in **b**. Note that in **e** the flow strength, which is given by the colour, represents the relative radially integrated flux *R*(*θ*)/*R*(*θ*)_max_, where 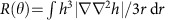, *R*(*θ*)_max_ is the maximum value of this across all *θ* and *h* is the local drop height (see Methods for more details).

The focus now moves to investigating the bulk flow within the drop. Figure 3a illustrates the typical stain left by a spherical drop of coffee evaporating under quasi-isothermal conditions once again. The pattern is characterized by a higher accumulation of coffee particles near the contact line evenly distributed in the azimuthal direction. This is the well-known ‘coffee ring effect'^4^,^5^. When the contact area is triangular (Fig. 3b), however, substantially darker regions emerge at the corners of the contact area. Another example corresponding to a square configuration is shown in Fig. 3c. Deegan *et al*.^4^,^5^ also noticed that curvature influenced the deposition in irregular drops, but the authors restricted their study to the spherical geometries in order to demonstrate that the coffee ring effect in isothermal conditions is caused by the continuity flow towards the contact line resulting from the non-uniform evaporation flux along the drop interface in the radial direction. The fact that non-spherical drops lead to darker stains at the corners is, therefore, an indirect experimental evidence that the local evaporation flux is no longer constant along the contact line; it is larger at the corners than along the rest of the perimeter, effectthat we already observed by analysing the evaporation kinetics. The direct mapping between **J** (Fig. 2c) and the particle deposition pattern is evident (Fig. 3b). Consequently, one also expects preferential continuity-induced currents towards these regions being developed in the bulk flow of the drop.

These conjectures are corroborated by our numerical simulations of a low-order model developed to describe the spatiotemporal evolution of the droplet and particle concentration field. The solution strategy, explained in detail in the Methods section, involves the division of the spatial domain into two regions. In the ‘inner' region, occupied by the drop, we use lubrication theory in order to reduce the governing Stokes equations to two partial differential equations for the drop height, *h*, and the depth-averaged particle concentration, *φ*. A diffusion-limited model is used to predict the vapour concentration in the ‘outer' region, from which we can derive the evaporative flux **J**; note that no scale separation is assumed in this region. This ultimately gives rise to a pair of coupled evolution equations for the interface and particle concentration (the details are in the Methods section), which were solved numerically starting from an initial state of a triangular drop. We find that evaporation is indeed significantly enhanced at the apices, which lead to a preferential particle accumulation in these regions, as shown by the resultant residue distribution illustrated in Fig. 3d. The locally enhanced evaporation flux results in an intensified mass deficit in more curved regions, which is rectified by a resultant capillary-driven flow that convects the particles towards the corners, as shown in the flow field depicted in Fig. 3e. These results are in accordance with previous theoretical predictions on the residue growth in an angular region^22^. The non-uniform coffee ring effect is, thus, the result of locally varying evaporation fluxes whose origin has been described in detail above.

### Drops of binary mixtures

We conclude by examining the dynamics of sessile drops of binary mixtures. Using PIV, Christy *et al*.^14^ reported the existence of three stages in the evaporation process of spherical water–ethanol drops. Further insights were given by Bennacer and Sefiane^23^. These authors found that the drop flow was very complex initially; multiple vortices with random orientation emerged and disappeared while the average absolute vorticity remained constant. A second transition stage followed in which there was a spike in the radially outward flow accompanied by an exponential viscous-driven decay of the vorticity. In the final stage, the outward flow was identical to that found in pure water drops. Qualitatively similar continuous mixing by distinct Marangoni flows has been recently observed in evaporating drops of whiskey^15^. It should be noted that the evaporation of the binary-mixture drops features a solutal-thermocapillary flow, that is, *σ*=*σ*_0_−*γ*_*T*_(*T*−*T*_0_)−*γ*_*c*_(*c*−*c*_0_) where *γ*_*T*_=−∂*σ*/∂*T*, *γ*_*c*_=−∂*σ*/∂*c*, and *c* here is the ethanol concentration in the liquid; normally, 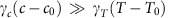. Some preliminary insights into the dynamics of this initially chaotic system were provided by Sternling and Scriven^24^ for planar films, but research into this subject remains on going.

**Figure 4:**
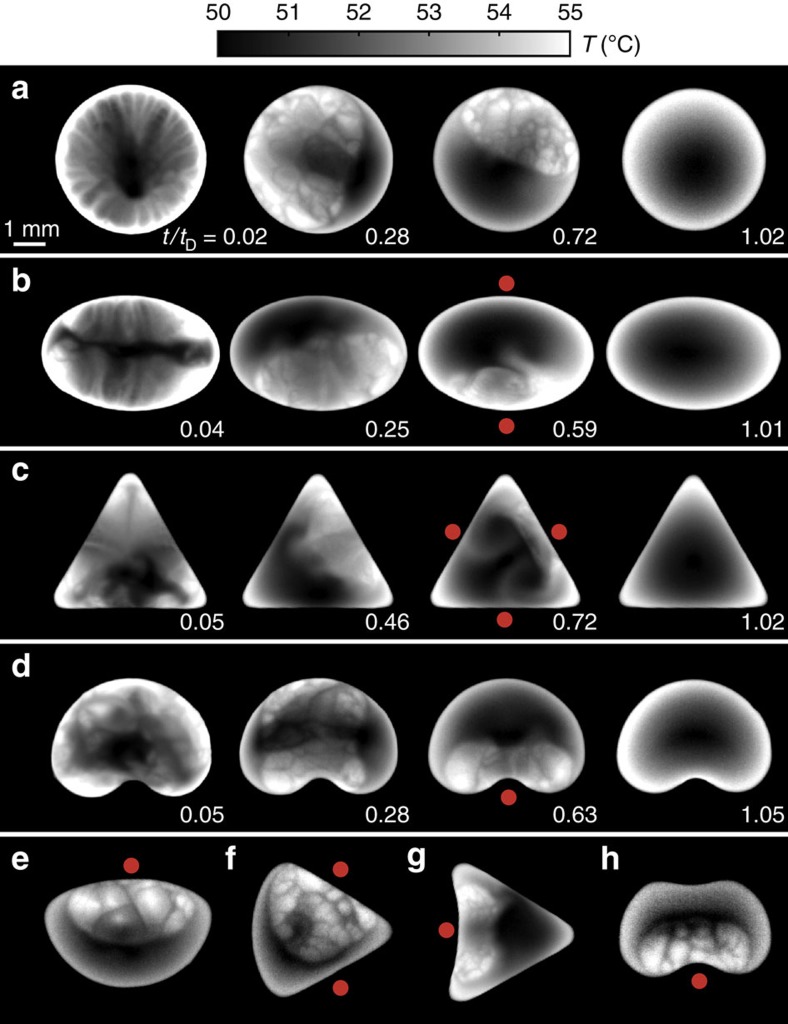
Flow dynamics of binary-mixture drops. Infrared images (top view) illustrating the ethanol depletion occurring in evaporating binary-mixture drops (ethanol–water) with different shapes. In all cases the initial volume is *V*=7 μl (25% vol ethanol, 75% vol distilled water), *P*=15 mm and *T*_w_=55 °C. The interfacial ‘turbulence' gives a measure of the ethanol concentration. The depletion time is *t*_D_=55.2 s±6.9%. In non-spherical drops, the segregation point always corresponds to the region where the contact-line curvature *κ*_cl_ is minimum; the red dots mark these locations. (**a**) *κ*_cl_=0.42 mm^−1^, (**b**) 0.24⩽*κ*_cl_⩽0.79 mm^−1^, (**c**) 0⩽*κ*_cl_⩽2.5 mm^−1^, (**d**) −1.87⩽*κ*_cl_⩽0.78 mm^−1^. (**e**–**h**) Other shapes with different principal curvature combinations (namely, positive–positive, zero–positive, negative–zero, negative–negative). Sample movies available in the Supplementary Materials (See Supplementary Movies 1 and 2).

We revisited these experiments using infrared thermography and also considered non-spherical geometries (see Fig. 4), which allowed us to observe that there is a spontaneous segregation of the more volatile component (ethanol) during its evaporation. Consistent with previous lines of work^14^,^23^, the thermal field exhibited a very intricate and agitated evaporation-driven interfacial motion in the initial stages. Total calm was observed when the chamber was saturated, that is, any phase change stopped, which demonstrates that evaporation is the driving mechanism. Interestingly, we found that this chaotic thermal motion covered the interface completely at the beginning, but gradually reduced its area of activity. The motion observed in the unoccupied regions was minimal, similar to what one would expect for pure water. Eventually, this area of thermal agitation disappeared completely, giving way to the characteristic motionless temperature field of pure water drops.

More interestingly, the area of interfacial turbulence maintained its integrity and always tended towards a region adjacent to the contact line (Fig. 4). No preferential location on the contact line was observed in spherical drops. In non-spherical drops, however, geometry dictated that the area of interfacial turbulence always moved to the region of minimum *κ*_cl_. For instance, in a drop with an elliptical contact area (positive–positive *κ*_cl_), the preferred region was always either of the two ends of the short axis (see Fig. 4b). In a triangular drop, where the curvature is large at the corners and zero elsewhere (positive-zero *κ*_cl_), the preferred region was any of the three flat sides (Fig. 4c). In a drop with positive-negative *κ*_cl_, such as the kidney shape shown in Fig. 4d, the preferred region was always concentrated at the dimple. Other more exotic shapes corroborating this observation are presented in Fig. 4e–h.

We rationalize the predefined spatial segregation in terms of the local evaporation flux distribution. The coffee ring effect and the scaling law previously discussed denote the enhanced evaporation associated with high-curvature regions. Base corners (Fig. 2c) are the areas where the evaporative flux is maximal and the thermal turbulence is more vigorous, given that this is driven by phase change. Regions with maximum *κ*_cl_ are thus optimal points for the evaporation of the more volatile component (ethanol). Accordingly, evaporation is lowest where *κ*_cl_ is minimal and, therefore, these are the areas where the last traces of ethanol will be found as this is depleted. This is exactly what our experiments have shown. We examined the role of temperature (*T*_w_=40, 55 and 60 °C) and solute concentration (*c*=10, 25 and 50% vol ethanol), and observed that the vigour of the flow increased with *T*_w_, and the duration of the interfacial ‘turbulence' with *c*, but the flow was otherwise qualitatively similar. Qualitatively, the same solutal dynamics were observed with non-heated drops.

## Discussion

We have investigated the flow dynamics and evaporation kinetics of non-spherical drops by means of experiments and theoretical modelling. We revisited the coffee ring effect and showed that apices in the contact area lead to larger particle depositions and preferential continuity-driven currents in the bulk of the drop towards them. The study of the evaporation kinetics revealed an accordingly varying local evaporation flux distribution, that is, the flux is maximum in areas of large contact-line curvature and *vice versa*. A universal scaling law for the overall evaporation rate including shape effects was deduced, which denotes the enhanced evaporation of curved interfaces. Infrared examination of the dynamics of evaporating ethanol–water drops also revealed a spontaneous segregation of the more volatile component, ethanol. Interestingly, the segregation point was found to be dictated by the drop geometry and always corresponded to the area where the contact-line curvature was minimum. All these previously unexplored phenomena are explained in terms of the inverse relationship existing in non-spherical drops between the contact-line curvature and the contact-angle distribution. In view of our results, we believe that the drop geometry emerges as a robust and versatile controlling mechanism for the solute deposition, flow and mixing dynamics, and evaporation kinetics of sessile drops. Finally, we also show in the Supplementary Note 1 that the critical threshold for the thermocapillary instability of heated ethanol drops is spatiotemporally-varying; maximum (minimum) contact-line curvature regions were found to be the most unstable (stable) areas.

## Methods

### Experimental techniques

**Figure 5:**
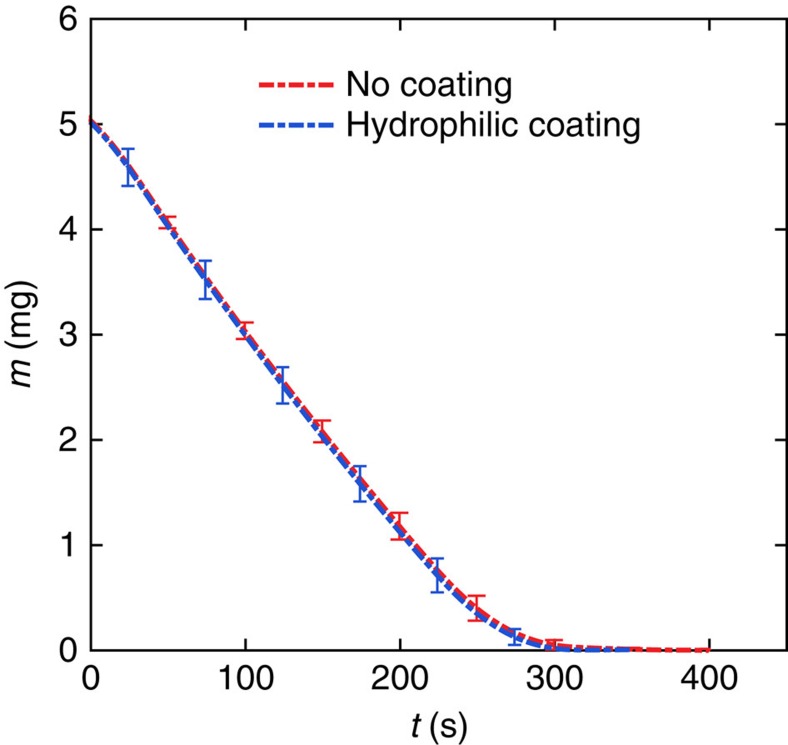
Effect of hydrophilic coating on evaporation of ethanol drops. Instantaneous mass of an ethanol drop with a circular contact area demonstrating that the hydrophilic coating did not have any effect in the evaporation kinetics of the sessile drops under consideration.

**Figure 6:**
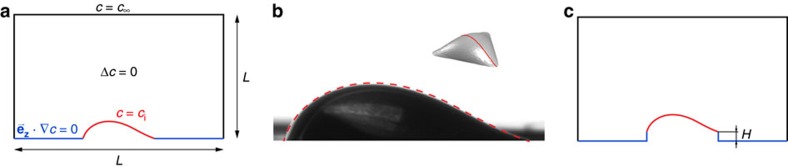
3D numerical problem. (**a**) Two-dimensional schematic. (**b**) Comparison of the experimental drop shape and the geometry calculated with Surface Evolver. (**c**) Problem investigating the effect of the pedestal height. Note that the schematics are not to scale and the simulations are in 3D.

The height of the thin pedestals machined in the aluminium disk was *H*=0.1±0.05 mm. Simulations as those described in Fig. 6c were conducted to assess the effect of the pedestal height. For the intermediate triangular geometry (*P*=15 mm), the maximum volume (*V*=7 μl), and varying *H*, we found that the resulting evaporation rate 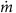 increases minimally with *H*. The difference in 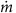 between *H*=0 and *H*=0.2 mm is <2%, which is negligible for the purposes of this investigation. One can, thus, assume that the drop is resting on a perfectly flat substrate. In cases in which the fluid was pure water, the top surface of the polished aluminium substrate was pretreated with a hydrophilic coating (Lotus Leaf HydroPhil-S) to improve the wettability and ensure precise and uniform pinning along the contact line. Several tests were performed to ensure that the solid particles from the coating remained on the substrate after the drop evaporation. The evaporation rate was also measured for drops placed on coated and uncoated surfaces. The comparison, showing no difference, is presented in Fig. 5. Finally, we also used the infrared camera to examine the thermal field in drops with and without the pedestal and hydrophilic coating. Once again, no difference was observed in either case.

### Numerical algorithms

*Evaporation kinetics*. We consider only the gas phase to investigate the evaporation kinetics of non-heated sessile drops of pure fluids. Under these conditions and for liquids with relatively low volatility as those considered in this work, it can be shown that the evaporation timescale is significantly larger than the timescale of species diffusion in the gas^1^. The process is, thus, quasi-stationary and can be accurately approximated by solving the problem of steady-state isothermal diffusion of vapour in the non-condensable gas (air). A schematic of the model is presented in Fig. 6a. We thus solve


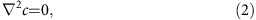


in the gas with the boundary conditions given in Fig. 6a. Here *c* is the vapour mass concentration in the air. The domain is a cube of size *L* × *L* × *L* (where computations were performed to ensure *L* is large enough to minimize end effects without prohibitive computational cost) and the drop shape is introduced through the geometry of the lower boundary. Along this curved surface, the vapour concentration is constant and equal to the saturation concentration^20^, that is, *c*=*c*_*i*_ where *c*_*i*_ is the interface vapour concentration calculated with Raoult's law and the saturation pressure at room temperature. The no-penetration condition **e**_**z**_·∇*c*=0 is implemented in rest of the lower boundary. The vertical and top boundaries are assumed to be very far from the drop and, therefore, the vapour concentration there is equal to that in the ambient value, that is, *c*=*c*_∞_. This closes the boundary value problem. Since surface tension dominates gravity in these drops, the drop interface shape is the result of the surface energy minimization of the system. We thus use Surface Evolver^21^ to calculate these. As in the experiments, the contact line and the volume are prescribed, and therefore the resulting geometry is unique. A comparison between the experimental drop geometry and that calculated with Surface Evolver for the same contact area and volume is presented in Fig. 6b.

**Figure 7:**
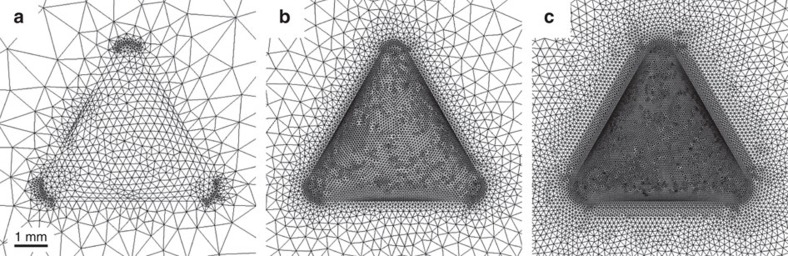
Examples of grids around the drop with increasing element density considered in the mesh dependency test. Bottom view. According to Table 1: (**a**) coarsest, (**b**) fine and (**c**) finest.

The evaporation flux at the drop surface is


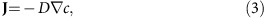


where *D* is the air–vapour molecular diffusion coefficient. It follows that the evaporation rate is defined as


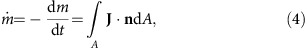


where *A* is the drop surface area. Given the simplicity of the governing equation and the complexity of the 3D domain, open-source (OpenFoam) or commercial (Comsol Multiphysics) codes are the most efficient solvers for this problem. We choose to use the latter, which allows finite element method (FEM) computations with unstructured tetrahedral meshing of the domain with finer resolution near the drop. The results of a mesh dependency test are presented in Table 1 and Fig. 7. Finally, we investigate the effect of the domain size *L*. With the chosen grid resolution, we conduct the same simulation for increasing size of the cubic domain, namely *L*=100, 150, 200 and 250 mm. The resulting evaporation rates decrease asymptotically to some value of 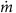. As *L* increases, the variation in 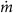 tends to zero. The evaporation rate for *L*=200 mm is only 1.2% larger than that for 250 mm and, therefore, assumed good enough. *L*=200 mm is, thus, the domain size chosen for the rest of the computations.

*Particle deposition*. We work in Cartesian coordinates (*x*, *y*, *z*) with a liquid drop of constant viscosity *μ* and density *ρ* deposited on an ultrathin precursor film of negligible volume, resting on a horizontal substrate located at *z*=0. Above is a hydrodynamically passive gas region. Following the standard approach, the evaporation from the precursor film is taken to be negligible with respect to the macroscopic evaporation of the sessile drop. The liquid is assumed to be sufficiently viscous that the hydrodynamic portion of the problem is governed by the Stokes equations subject to no-slip at the lower wall, and appropriate stress conditions at the interface,


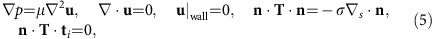


where *σ* is the (constant) surface tension and *i*=1, 2 correspond to the two orthogonal tangent vectors to the surface. Note that we have no disjoining pressure so that the drop will be perfectly wetting. Following Espin and Kumar^25^, we allow the wall to be covered with a precursor film of uniform thickness except for a small ‘dimple' at the contact line in order to pin the edge of the drop there. Thus, the wall is located at *z*=*ζ*(*x*, *y*) with *ζ*=−*a* exp((*r*−*r*_c_)^2^/2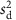), where *a* is the ‘depth' of the dimple, *s*_d_ prescribes its width, 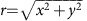 is the radial distance from the point on the substrate under the apex of the drop and *r*_c_(*θ*) is chosen to prescribe a triangular contact line. We take *a*=0.05 and *s*_d_=0.02. As found by Espin and Kumar^25^ this introduces a local minimum in the film thickness, which is taken to be the contact line, with the precursor film lying outside in which evaporation is switched off by setting *c*_z_=0.

The droplet is taken to contain nanoparticles of density *φ*_ρ_, which are assumed to follow an advection–diffusion equation with diffusion coefficient *D*_l_ subject to no-flux at the wall and interfacial diffusion flux due to concentration variation associated with evaporation, respectively,


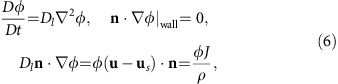


where **u**_s_ is the velocity of the interface.

As in the previous section, we use a diffusion-limited model for the evaporative effects, so that the vapour mass concentration *c* still satisfies Laplace's equation, subject to equality with the ambient value *c*_∞_ in the far field, *c*=*c*_*i*_ on the interface and *c*_z_=0 at the lower wall. This effectively describes a fully 3D problem in non-rectilinear domain including mixed boundary conditions. The flux *J* is then given by


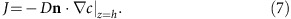


In order to make analytical progress, we exploit the fact that the drop is slender, and we divide the domain into two regions: an ‘inner' and an ‘outer' one. In the ‘inner' region, which corresponds to the body of the drop, we non-dimensionalize using the substitutions 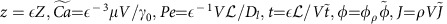, where 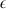 is a slenderness parameter given by the aspect ratio of the drop. This describes the scaled ‘inner' region in the body of the drop where the scaled variable 0⩽*Z*⩽*h* is appropriate. In the ‘outer' region, which corresponds to that occupied by the vapour, we retain the unscaled *z* coordinate. We note that, as viewed from the ‘outer' region, the interface is located at *z*=0+*O*(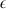). Dropping the tilde decoration, this ultimately leads to the governing equations^26^,


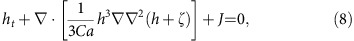



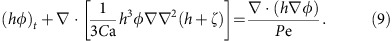


All that remains is to discern the form of the vapour mass concentration *c* (whence *J*). It is governed by Laplace's equation ∇^2^*c*=0 subject to *c*→0 as *z*→∞ (where we have subtracted off the irrelevant quantity *c*_∞_) and the mixed boundary condition


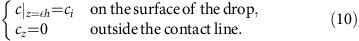


This can be simplified as follows. First, we note that this is posed in terms of the unscaled outer variable *z*. To leading order in 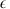, 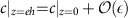, so that the first of these conditions can be evaluated at *z*=0. This is in accordance with the fact that the interface is located at *z*=0 at leading order as viewed from the ‘outer' region. Second, rather than attempting to evaluate the problem as a Laplace problem subject to a mixed boundary condition, we follow the approach of Popov^11^ and re-pose it as the solution to the Poisson problem


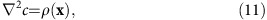


across all space. If *ρ* is any even function of *z*, the second condition of (10) is satisfied automatically. Therefore, in order to satisfy the first boundary condition we simply take


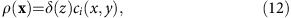


inside the contact line. The solution of the Poisson problem is given by a convolution with the appropriate Green's function^27^, so that


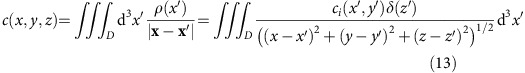



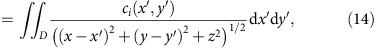


where *D* is the domain of the droplet. Finally, we note that to leading order in 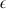, 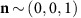 so that *J* can be expressed as


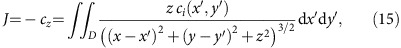


evaluated at the interface by quadrature.

**Figure 8:**
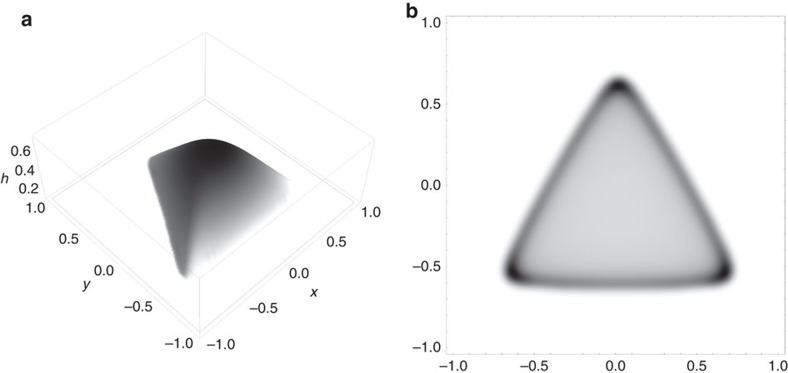
Particle deposition in triangular drops. (**a**) Initial triangular state of the drop. (**b**) Resultant residue for *C*a=0.1, *P*e=10^3^, demonstrating accumulation at the corners as expected.

**Figure 9:**
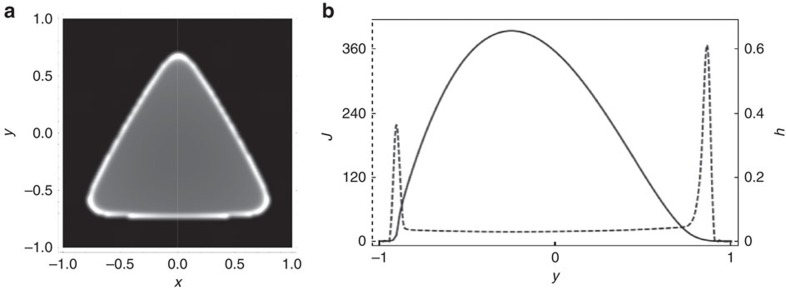
Local evaporation flux. Parameter values are as in Fig. 8. (**a**) Two-dimensional plot of the flux, *J*; (**b**) overlaid height (solid line) and flux (dashed) line corresponding to dashed white line in **a**, demonstrating the enhanced flux nearer the apices. The parameter values are the same as those used to generate Fig. 8.

We initialize our problem with the triangular profile shown in Fig. 8a. We solve it be using an semi-implicit, operator-splitting strategy based on the method (*pL*_1_) of Witelski and Bowen^28^. The mesh and time step are varied to ensure convergence. We find that a typical flux profile is as given in Fig. 9. In Fig. 9b, we overlay a plot of the drop height over the flux in the *x*=0 plane, shown by the dotted line in Fig. 9a, which illustrates clearly the enhanced evaporative flux close to the apices. A combination of contact-line pinning and continuity drive capillary-induced flow towards the corners, convecting particles towards these apices. Upon complete evaporation, Fig. 8b demonstrates the anticipated resultant spatial inhomogeneity in the residue distribution.

### Data availability

The data that support the findings of this study are available from the corresponding author upon reasonable request.

## Additional information

**How to cite this article:** Sáenz, P. J. *et al*. Dynamics and universal scaling law in geometrically-controlled sessile drop evaporation. *Nat. Commun.* **8,** 14783 doi: 10.1038/ncomms14783 (2017).

**Publisher's note**: Springer Nature remains neutral with regard to jurisdictional claims in published maps and institutional affiliations.

## Supplementary Material

Supplementary Information Supplementary Note, Supplementary Figure and Supplementary References

Supplementary Movie 1 Infrared video showing the alcohol segregation in an ethanol-water sessile drop with an elliptical contact area (P = 15 mm) evaporating on a heated metallic substrate held at constant temperature (Tw = 55 ℃).

Supplementary Movie 2 Infrared video showing the alcohol segregation in an ethanol-water sessile drop with a kidney-shaped contact area (P = 15 mm) evaporating on a heated metallic substrate held at constant temperature (Tw = 55 ℃).

Supplementary Movie 3 Infrared video showing the spontaneous development of thermocapillary instabilities in a spherical ethanol sessile drop evaporating on a heated metallic substrate held at constant temperature (Tw = 40 ℃).

Supplementary Movie 4 Infrared video showing the spontaneous development of thermocapillary instabilities in an ethanol sessile drop with a triangular contact area (P = 15 mm) evaporating on a heated metallic substrate held at constant temperature (Tw = 40 ℃).

## Figures and Tables

**Table 1:** Mesh dependency test carried out for a drop of ethanol with triangular contact area and *P* **=**15 mm and *V* **=**7 **μ**l.

	**Coarsest**	**Coarser**	**Coarse**	**Fine**	**Finer**	**Finest**
Max. Elm. size—drop surface (mm)	0.25	0.15	0.10	0.08	0.07	0.06
Max. Elm. size—gas (mm)	5.5	4.8	4.4	4.1	3.8	3.6
Max. growth rate	2.0	1.5	1.4	1.3	1.2	1.1
Number of tetrahedral Elm. (millions)	0.83	1.26	1.67	2.11	2.75	3.85
Evaporation rate (mg s^−1^)	0.01764	0.01808	0.01839	0.01846	0.01847	0.01856
Variation with respect to ‘finest' (%)	−4.96	−2.59	−0.92	−0.54	−0.48	0.00

Elm, element; Max, maximum.

Illustrations of some of these meshes are depicted in Fig. 7. The mesh ‘fine' is chosen for the rest of the simulations, given the negligible deviation in its resulting evaporation rate.
