# Supplementary material for: Dynamics and universal scaling law in geometrically-controlled sessile drop evaporation
Source: Nat Commun. 2017 Mar 15;8:14783. doi: 10.1038/ncomms14783 (PMC5355953; doi:10.1038/ncomms14783)
Supplement: Supplementary Information — Supplementary Note, Supplementary Figure and Supplementary References [file ncomms14783-s1.pdf]

Supplementary Note 1

As part of this work, we also examined to the vigorous thermocapillary instabilities appearing spontaneously in heated drops of ethanol. This type of instabilities was initially studied in liquid layers subjected to vertical[1;2] and lateral[3] heating. In sessile drops, this phenomenon was first observed by Sefiane *et al.*[4] It is important to note that the bulk flow is now completely dominated by thermal Marangoni convection instead of evaporation-driven continuity. The dominant nonlinearities of this problem preclude the explanation of its evaporation kinetics in simple terms (quasi-steady diffusion) as before, and a significantly more complex two-phase model[5] is necessary for its investigation. For most pure liquids, such as ethanol or water, surface tension σ decreases monotonically with temperature T, i.e. $\sigma = \sigma_0 - \gamma_T(T - T_0)$ where $\gamma_T = -\partial\sigma/\partial T$. Hence, the bulk flow in heated sessile drops usually consists of warmer liquid from the contact-line region being convected by surface tension gradients along the interface towards the top, where evaporative cooling maintains it at a lower temperature. The strength of this flow is normally characterized by the Marangoni number $Ma = \gamma_T H^2 \Delta T/(\mu\alpha L)$ where H and L denote the characteristic vertical and radial length scales, $\Delta T = T_w - T_{apex}$ is the temperature difference between the substrate and the apex, μ is the viscosity, and α the thermal diffusivity.[4] For the ethanol drops shown in Fig. 1, typically $\Delta T \sim 5$°C, $H \sim 1\,\mathrm{mm}$, $L \sim 2\,\mathrm{mm}$, and therefore $Ma \sim 3.5 \times 10^3$. In the spherical configuration (Fig. 1a), the initial temperature distribution remained concentric until the interfacial thermal gradient in the radial direction crossed a critical Marangoni threshold, after which the drop developed petal-like thermal fluctuations that moved in the azimuthal direction. The shape, size, wavenumber, and direction of propagation of these instabilities change as the drop evaporates.

In the non-spherical case, illustrated with the triangular geometry (Fig. 1d), important differences are readily apparent. Firstly, before any stability was observed, the flow was arranged in a well-defined three-fold structure with cold bands connecting the top with the base apices. If one thought of this drop as a triangular pyramid, the cold bands would correspond to the pyramid's edges meeting at the top. This well-structured flow is rationalized in terms of the varying strength of the Marangoni flow along the azimuthal direction, see Fig. 1h. For the same ΔT, the thermocapillary flow ranges between two limiting sections, namely $\overline{P_1O}$ and $\overline{P_2O}$. We compute the interface path length L and the line-averaged drop height $\overline{H} = \int_L H \mathrm{d}L / \int_L \mathrm{d}L$ along these two sections and calculate the corresponding path-related Marangoni numbers, i.e., $Ma_{\overline{P_1O}}$ and $Ma_{\overline{P_2O}}$, respectively. The ratio between these is presented in Fig. 1i; L is smaller and $\overline{H}$ larger along the $\overline{P_1O}$ path. For the triangular drop $Ma_{\overline{P_1O}} >$

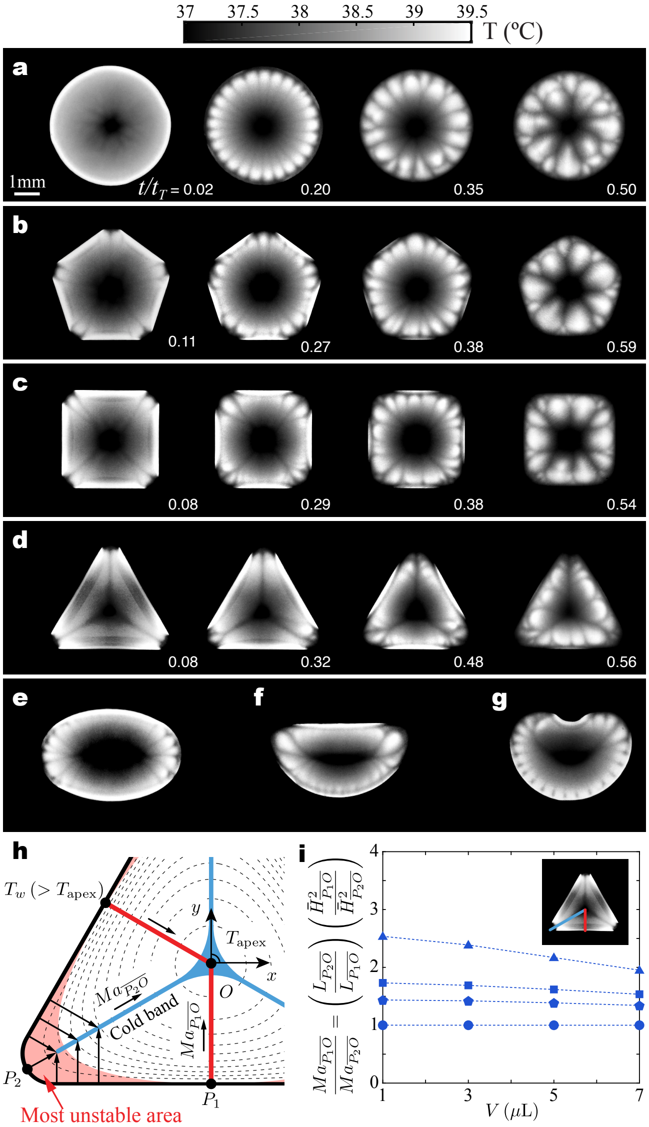

Supplementary Figure 1: Thermocapillary flow and instabilities in heated ethanol drops. Interface temperature distribution recoded via infrared thermography (top view) illustrating the spatially nonuniform onset and growth of thermocapillary instabilities in evaporating sessile drops of pure ethanol with different shapes. Here, $P = 15$ mm and the initial volume $V = 7\ \mu$L. The substrate is held at constant temperature $T_w = 40°$C. The drop lifetime is: (a) $t_T = 99.4$ s $\pm$ 2.5%, (b) 104.1 s $\pm$ 5.1%, (c) 114.9 s $\pm$ 3.4%, (d) 119.7 s $\pm$ 5.0%. In non-spherical drops, the thermocapillary instabilities always grow first (last) where the contact-line curvature κ_{cl} is maximum (minimum). The contact-line curvature is $\kappa_{cl} = 0.42$ mm^{-1} in (a) and ranges between $0 \leq \kappa_{cl} \leq 2.5$ mm^{-1} in (b-d). Panels (e-g) show other drops with different principal curvature combinations (namely, positive-positive, zero-positive, negative-positive). Sample movies available in the supplementary materials. (h) Schematic of the driving mechanism of the interfacial flow in a triangular drop. (i) Relative strength of the driving force (Marangoni number) along the $\overline{P_1O}$ ($Ma_{\overline{P_1O}}$) and $\overline{P_2O}$ ($Ma_{\overline{P_2O}}$) principal sections shown in (h) and equivalent sections in square and pentagonal drops. The markers denote the drop shapes.

$2Ma_{\overline{P_2O}}$. Note that the disparity between limiting Ma numbers decreases asymptotically to unity for increasing number of sides. Hence, the amount of warm fluid transported from the contact line along $\overline{P_2O}$ is much smaller than that transported along $\overline{P_1O}$, which explains the emergence of the cold bands. These bands were not observed in water droplets.

The second key observation was that the thermocapillary instabilities in non-spherical drops always grew first (last) in the regions where κ_{cl} was largest (smallest), and did not fill the contact-line at once as in the circular case (Fig. 1b-d). This finding was also confirmed with more complicated shapes (Fig. 1e-g). Note that in the kidney shape (Fig. 1g), the only region free of instabilities is in the dimple, where $\kappa_{cl} < 0$. Since θ is significantly smaller around the corners, these areas are more similar to thin films while the interface slope is high elsewhere. Thinner films are known to be more unstable to thermocapillary instabilities due to the stabilizing effect of gravity.[1;3;6;7] This can be characterized by the dynamic Bond number $Bo_d = \rho g \beta H^2/\gamma_T$, where β is the coefficient of volumetric expansion, which is proportional to the thickness squared. The same effect explains the final difference of note: it took the instabilities a significantly longer time to fill the drop completely in the non-spherical configurations. For instance, in the spherical drop the instabilities were present everywhere by $t/t_T = 0.20$ while in the triangular configuration this did not occur until $t/t_T = 0.56$. Here, t is the time, and t_T the drop lifetime. Riley & Neitzel,[8] for example, studied this effect in laterally-heated films in terms Bo_d and found that the critical Marangoni number Ma_c grew exponentially with very small variations of Bo_d. Since our drops had the same P and initial V, and the circle is the geometrical shape that maximizes the closed area, the resulting drop height in deformed drops was always larger than in the spherical configuration of the same P. For $P = 15\,\text{mm}$ and $V = 7\,\mu$L, we found that $Bo_d^{\triangle} = 0.11$ and $Bo_d^{\bigcirc} = 0.06$. In general, $Bo_d^{\triangle} \approx 2Bo_d^{\bigcirc}$ for the same volume. According to the experiments by Riley & Neitzel,[8] this means that Ma_c must be significantly larger ($\sim 35\%$) in the triangular configuration, which explains the observed delay in the instabilities' growth.

Supplementary References

[1] J. R. A. Pearson. On Convection Cells Induced by Surface Tension. *J. Fluid Mech.*, 4(5):489–500, (1958).

[2] L. E. Scriven and C. V. Sternling. On Cellular Convection Driven by Surface-Tension Gradients - Effects of Mean Surface Tension and Surface Viscosity. 19(3):321–340, (1964).

[3] M. K. Smith and S. H. Davis. Instabilities of Dynamic Thermocapillary Liquid Layers. Part 1. Convective Instabilities. *J. Fluid Mech.*, 132(Jul):119–144, (1983).

[4] K. Sefiane, J. R. Moffat, O. K. Matar, and R. V. Craster. Self-excited hydrothermal waves in evaporating sessile drops. *Appl. Phys. Lett.*, 93(7):74103, (2008).

[5] P. J. Sáenz, K. Sefiane, J. Kim, O. K. Matar, and P. Valluri. Evaporation of sessile drops: a three-dimensional approach. *J. Fluid Mech.*, 772:705–739, (2015).

[6] P. J. Sáenz, P. Valluri, K. Sefiane, G. Karapetsas, and O. K. Matar. Linear and nonlinear stability of hydrothermal waves in planar liquid layers driven by thermocapillarity. *Phys. Fluids*, 25(9):094101, (2013).

[7] P. J. Sáenz, P. Valluri, K. Sefiane, G. Karapetsas, and O. K. Matar. On phase change in Marangoni-driven flows and its effects on the hydrothermal-wave instabilities. *Phys. Fluids*, 26(2):024114, (2014).

[8] R. J. Riley and G. P. Neitzel. Instability of thermocapillary-buoyancy convection in shallow layers. Part 1. Characterization of steady and oscillatory instabilities. *J. Fluid Mech.*, 359:143–164, (1998).
